# Cryo-EM structures reveal variant Tau amyloid fibrils between the rTg4510 mouse model and sporadic human tauopathies

**DOI:** 10.1038/s41421-023-00637-w

**Published:** 2024-03-07

**Authors:** Wanbing Zhao, Kaien Liu, Yun Fan, Qinyue Zhao, Youqi Tao, Mengwei Zhang, Linhua Gan, Wenbo Yu, Bo Sun, Dan Li, Cong Liu, Jian Wang

**Affiliations:** 1 Department of Neurology and National Research Center for Aging and Medicine & National Center for Neurological Disorders, State Key Laboratory of Medical Neurobiology, Huashan Hospital, Fudan University, Shanghai, China; 2 Interdisciplinary Research Center on Biology and Chemistry, Shanghai Institute of Organic Chemistry, Chinese Academy of Sciences, Shanghai, China; 3 Bio-X Institutes, Key Laboratory for the Genetics of Developmental and Neuropsychiatric Disorders (Ministry of Education), Shanghai Jiao Tong University, Shanghai, China; 4 School of Life Science and Technology, ShanghaiTech University, Shanghai, China; 5 Zhangjiang Institute for Advanced Study, Shanghai Jiao Tong University, Shanghai, China; 6 WLA Laboratories, World Laureates Association, Shanghai, China; 7 State Key Laboratory of Chemical Biology, Shanghai Institute of Organic Chemistry, Chinese Academy of Sciences, Shanghai, China

**Keywords:** Cryoelectron microscopy, Protein aggregation

Dear Editor,

Tau amyloid accumulation is a prevalent pathological feature observed in various neurodegenerative diseases such as Alzheimer’s disease (AD), corticobasal degeneration (CBD), globular glial tauopathy (GGT), familial frontotemporal lobar degeneration (FTLD), chronic traumatic encephalopathy (CTE), Pick’s disease (PiD), argyrophilic grain disease (AGD), and progressive supranuclear palsy (PSP), collectively termed tauopathies^1,2^. In these diseases, Tau amyloid fibrils accumulate intracellularly, leading to an array of pathological outcomes, from prion-like propagation and protein homeostasis disruption to enhanced neuroinflammatory response^3^. Importantly, recent cryo-electron microscopy (cryo-EM) studies have revealed that Tau exhibits unique amyloid fibril structures in the brains of patients across different tauopathies, correlating with specific disease variants. This underscores the critical influence of individual Tau fibril structures in steering disease progression, dictating the clinical-pathologic presentation, and providing atomic and molecular fingerprints in classifying tauopathy subtypes^4,5^.

Considering the pivotal role of Tau aggregation in AD and other tauopathies, mouse models such as rTg4510 and PS19 overexpressing disease-associated Tau mutations have been developed and widely used to investigate the disease mechanism as well as for drug development^6,7^. These models manifest symptoms such as age-correlated memory loss, specific brain region Tau accumulation, and neuron degeneration, which effectively echo the clinical and pathological facets of tauopathies^8^. However, given the vast clinical diversity inherent to tauopathies, it is crucial to dissect the molecular specifics to ascertain which tauopathy subtypes these mouse models can truly replicate, especially concerning the structures of Tau amyloid fibrils present in these models^4,5^.

**Fig. 1:**
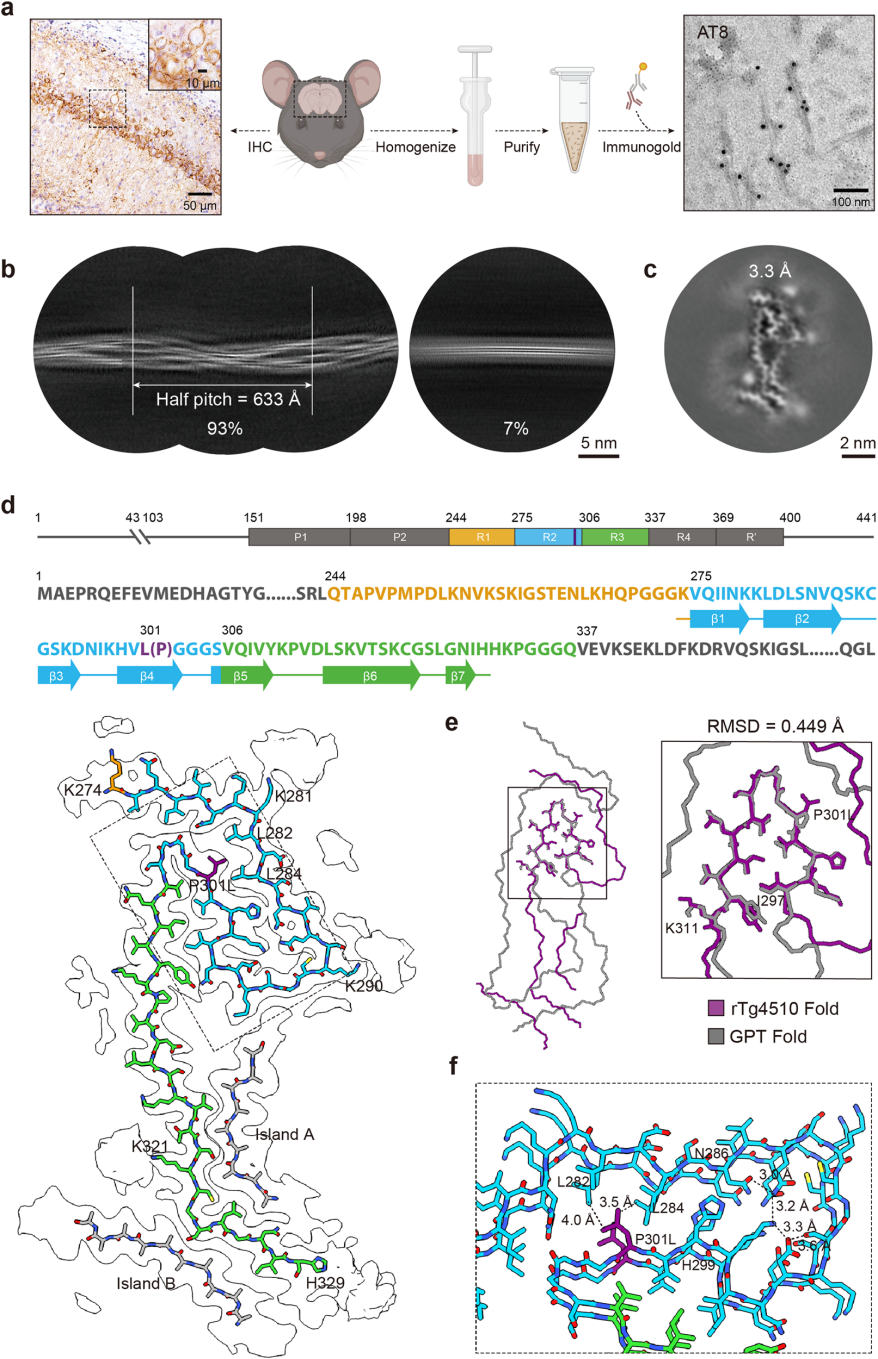
Cryo-EM structure of rTg4510 Tau fibril. **a** The workflow of the brain-derived rTg4510 Tau fibril purification and characterization. Immunohistochemistry staining of p-Tau (AT8) in the hippocampus of rTg4510 is shown on the left. Scale bar, 50 μm. The ex vivo Tau fibril was confirmed by immunogold negative-staining electron microscopy as shown on the right. Scale bar, 100 nm. **b** 2D class averages of rTg4510 Tau fibril (left) and minority untwist fibril (right). The percentages of the two types of ex vivo fibrils in the dataset and the half pitch of the rTg4510 Tau fibril are shown at the bottom. Scale bar, 5 nm. **c** 3D classification of the rTg4510 Tau fibril. Resolution of 3D reconstruction is indicated. Scale bar, 2 nm. **d** Top, diagram of human 0N4R Tau primary amino acid sequence. Residue 44 is followed by residue 103. All residues are numbered based on human 2N4R Tau isoform. N-terminal region, R4 region, R’ region, and C-terminal region are shown in black, with R1 region in orange, R2 region in blue and R3 in green. Middle, primary amino acid sequence of human 0N4R Tau with P301L mutation. Residues are colored as in top with P301L mutation indicated in purple. Thick connecting lines with arrowheads indicate β-strands. Bottom, atomic model of the rTg4510 Tau fibril overlaid with cryo-EM density map. Residues are colored as in top with islands A and B indicated in gray. **e** Comparison of the rTg4510 Tau with GPT fold (PDB:7P6A). The part using for comparison (residues 297–311) is zoomed in on the right panel. Local RMSD is indicated. **f** Zoom-in view of the corresponding region of (**d**, bottom). Distances of interactions between residues that stabilize the turn are indicated.

In this study, we sought to determine the cryo-EM structure of Tau amyloid fibrils in the rTg4510 mouse model. As one of the most widely used mouse models, rTg4510 utilizes the CamK2a promoter to overexpress 0N4R human Tau carrying the P301L mutation — one of the most common mutations of Tau associated with familial FTLD, in the mouse forebrain^9,10^. We initially studied 9-month-old rTg4510 mouse brains using immunohistochemistry and observed significant p-Tau pathology (Fig. 1a). We subsequently extracted amyloid fibrils from these brains using the sarkosyl extraction method^11^. An abundance of fibrils was obtained, and further verification using immunogold negative-stain electron microscopy with Tau-specific antibody confirmed that the fibrils are composed of Tau (Fig. 1a).

We then collected 5000 cryo-EM micrographs to solve the structures of these fibrils. Subsequent fibril picking and two dimensional (2D) classification revealed that ~93% of the 2D class averages demonstrate twists and can be attributed to an identical fibril species (Fig. 1b). After helical reconstruction, we obtained a three dimentional (3D) density map with an overall resolution of 3.3 Å (Fig. 1c and Supplementary Fig. S1). The fibril comprises a single protofilament with a crossover distance of ~633 Å (Fig. 1b and Supplementary Fig. S2). The helical twist between subunits is –1.36° and the helical rise is 4.78 Å (Supplementary Fig. S2 and Table S1).

Based on the high-resolution density map, we were able to unambiguously build a structure model for the ex vivo Tau fibrils (Fig. 1d). The fibril core encompasses residues 274–329 (residue numbering based on the 2N4R Tau), which include the C-terminal lysine of R1, the entire R2 and 24 amino acids of the succeeding R3 (Fig. 1d). This structure contains seven β-strands (β1–7), organized into a serpentine fold (Fig. 1d). Two elongated strip-like densities in the shape of peptide constituents, termed Islands A and B, are also evident. Island A aligns against R3, and connects with densities of R2 proximate to K290; Island B aligns against R3 as well on the other side, connecting with densities proximate to K321 (Fig. 1d). However, the absence of distinguishable side chain densities hindered precise identification. In addition to the structured core, three additional densities present adjacent to K281, K290, and K321, suggesting potential ubiquitination^12,13^ (Fig. 1d).

Based on the reported structures of ex vivo Tau fibrils extracted from human brains, eight distinct Tau folds, including AD, CTE, PiD, CBD, AGD, PSP, GGT, and GGT-PSP-Tau (GPT) folds, have been identified in diverse tauopathies^5^. Of note, these human brain Tau proteins contain no genetic mutations in their fibril structures. In contrast, the structure of the human P301L mutant Tau fibrils extracted from the rTg4510 mouse markedly diverges from these Tau folds (Fig. 1e and Supplementary Fig. S3). Yet, when overlayed with the five ex vivo 4R Tau fibril structures, including GPT, GGT, AGD, CBD and PSP folds, a part of the P301L Tau (residues 297–311) that displays a U-shape, exhibits a similar structure to them (Fig. 1e and Supplementary Fig. S3). Especially, this U-shaped motif in the P301L Tau closely resembles those in the GPT and GGT folds, with Root Mean Square Deviation (RMSD) (for Cα atoms) values of 0.449 Å and 2.269 Å, respectively^5^ (Fig. 1e and Supplementary Fig. S3d).

By comparing the P301L structure with the GPT structure, it is observed that the P301L mutation introduces a bulky hydrophobic side chain of Leu, which leads to steric hindrance against the interactions with H362 and P364 as seen in the GPT fold and preventing the attachment the C-terminal segment (residues 359–379) to the U-shaped motif (Supplementary Fig. S4). Instead, the P301L mutation establishes pronounced hydrophobic interactions with L282 and L284, which together with hydrogen bonding between the side chains of residues 286–299, attract the N-terminal segment (residues 274–299) to the U-shaped motif forming in the unique P301L fold (Fig. 1f and Supplementary Fig. S5).

Tau has been observed to form diverse fibril structures, each with a disease-specific conformation stemming from unique pathological conditions. Thus, Tau fibril structures have been suggested for the classification of tauopathy subtypes at the atomic level^5^. Our research uncovers that the widely used rTg4510 mouse model expressing FTLD-associated human P301L mutant Tau, develops amyloid fibrils whose structure is notably different from those in human brains diagnosed with non-hereditary tauopathies. This structural difference might stem both from the P301L mutation and the differing cellular contexts between human and mouse. Our finding suggests that the rTg4510 mouse model could not precisely mirror sporadic human tauopathies on atomic and molecular fronts. Hence, when probing into the disease mechanism of AD and other tauopathies, or in the development of Tau-targeting drugs, caution is advised in using this model^6,7^. Thus, for mechanistic study and drug development of tauopathies, it is important to refine animal models that not only resonate with human disease manifestations, but also mimic the specific Tau fibril structures associated with each disease.

The P301L Tau mutation was first identified in familial FTLD patients^10^. Intriguingly, a global GGT patient cohort study also identified P301L as the predominant pathogenic variation^14^. Our structural analysis revealed that although the P301L mutant Tau forms different fibrils from those extracted from human diseased brains, part of its fold, especially the U-shaped motif, generally presents in the human brain-derived Tau. This suggests that the rTg4510 mouse model might offer a partial reflection of the pathological traits and represent tauopathy in general. Nevertheless, in the rTg4510 mouse model, Tau adopts a distinctive fibril structure. It is yet to know whether this structure resembles the P301L Tau fibril in FTLD and GGT patients who carry this mutation. If so, the rTg4510 mouse might represent a precise model for these patients. Thus, future exploration is required to reveal the atomic structure of P301L Tau fibrils directly extracted from the brains of tauopathy patients who carry this mutation to corroborate the Tau fibril structure and validate the appropriateness of the rTg4510 mouse model.

### Supplementary information

Supplementary information, Figures and Tables
