# Supplementary material for: Cryo-EM structures reveal variant Tau amyloid fibrils between the rTg4510 mouse model and sporadic human tauopathies
Source: Cell Discov. 2024 Mar 7;10:27. doi: 10.1038/s41421-023-00637-w (PMC10917778; doi:10.1038/s41421-023-00637-w)
Supplement: Supplementary file 1 — Supplementary information, Figures and Tables [file 41421_2023_637_MOESM1_ESM.pdf]

LETTER TO THE EDITOR

Cryo-EM structures reveal variant Tau amyloid fibrils between the rTg4510 mouse model and sporadic human tauopathies

Wanbing Zhao[1¶], Kaien Liu[2¶], Yun Fan[1¶], Qinyue Zhao[3], Youqi Tao[3], Mengwei Zhang[1], Linhua Gan[1], Wenbo Yu[1], Bo Sun[4], Dan Li[3,5,6], Cong Liu*[2,7], Jian Wang*[1]

[1]Department of Neurology and National Research Center for Aging and Medicine & National Center for Neurological Disorders, State Key Laboratory of Medical Neurobiology, Huashan Hospital, Fudan University, Shanghai, 200040, China.

[2]Interdisciplinary Research Center on Biology and Chemistry, Shanghai Institute of Organic Chemistry, Chinese Academy of Sciences, Shanghai, 201210, China.

[3]Bio-X Institutes, Key Laboratory for the Genetics of Developmental and Neuropsychiatric Disorders (Ministry of Education), Shanghai Jiao Tong University, Shanghai, 200030, China.

[4]School of Life Science and Technology, ShanghaiTech University, Shanghai, 201210, China

[5]Zhangjiang Institute for Advanced Study, Shanghai Jiao Tong University, Shanghai, 200240, China.

[6]WLA Laboratories, World Laureates Association, Shanghai, 201203, China

[7]State Key Laboratory of Chemical Biology, Shanghai Institute of Organic Chemistry, Chinese Academy of Sciences, Shanghai, 200032, China

¶These authors contributed equally to this work.

*To whom correspondence should be addressed. E-mails: wangjian_hs@fudan.edu.cn; liulab@sioc.ac.cn

Materials and Methods

Transgenic mouse

rTg4510 mice express 0N4R human Tau with the frontotemporal dementia-associated P301L mutation[1] under the control of forebrain-specific calcium-calmodulin-dependent kinase II (CaMK2a) promoter on a mixed C57BL/6 X FVB background. Heterozygous mice develop hyperphosphorylated tau inclusions in the cortex and hippocampus by approximately 2.5 months[2]. Breeding pairs of rTg4510 mice were obtained from the Jackson Laboratory (024854, 015815). All the animal husbandry and related procedures were performed in accordance with the institutional guidelines and approved by the Animal Care and Use Committee of Fudan University.

Extraction of amyloid fibrils

Fibril extraction was essentially based on a published procedure[3]. In brief, PBS perfused and non-fixed mouse brain tissue was snap frozen in dry ice and stored at -80°C previous to experimentation. Whole mouse brain weight approximately 0.5g was thawed and homogenized for three times in 20-times volume (w/v) of extraction buffer (10 mM Tris-HCl, pH 7.5, 0.8 M NaCl, 10% sucrose,1 mM EGTA, 0.1% sarkosyl, PMSF and Cocktail). Subsequently, 10% sarkosyl was added to the homogenate to a final concentration of 2% and was incubated at 37°C for 1.5 h with manually shaking. The homogenate was centrifuged at 10,000 x g for 10min at 4°C and the resulting supernatant was further ultracentrifuged at 966,00 x g for 60 min at 4°C. After removal of the supernatant, the pellet was resuspended in extraction buffer (1 ml/g), followed by 3,000 x g centrifugation for 5 min at 4°C.

The supernatant was then diluted 3-fold in dilution buffer (50 mM Tris-HCl, pH 7.5, 0.15 M NaCl, 10% sucrose, 0.2% sarkosyl) and ultracentrifuged at 166,000 g for 1 h at 4°C. The sarkosyl-insoluble pellets (P4) were resuspended with 20 mM Tris-HCl (pH 7.4) and 50 mM NaCl (100μl/g), and was used for further negative staining, immunogold labelling and cryo-EM analysis.

Negative staining transmission electron microscopy (NS-TEM)

5 μL of P4 in resuspension buffer was loaded onto the glow-discharged 230 mesh carbon coated copper grids (Beijing Zhongjingkeyi Technology Co., Ltd.) and incubated for 45 s. Then the sample was removed by the filter paper.The grid was then washed with 5 μl dH2O and 5 μl of 1% (w/v) uranyl acetate (UrAc). Another 5 μl 2% w/v uranyl acetate was applied to stain the sample for 45 s. The sample imaging was accomplished by a Tecnai T12 microscope (FEI Company) operated at 120 kV.

Immuno-gold negative-staining electron microscopy

Immunogold negative stain electron microscopy was carried out as described[3]. Briefly, 5 μl of the final pellet containing the extracted Tau fibrils was loaded onto the glow-discharged 230 mesh carbon coated copper grids (Beijing Zhongjingkeyi Technology Co., Ltd.) for 2 min. The sample was washed twice with ddH2O and placed in blocking buffer (0.1%BSA in PBS) for 10 min at room temperature, following incubation with p-Tau antibody (MN1020, Invitrogen) at 1:50 for 2 h at room temperature. Then the grid was washed with ddH2O twice and was incubated with anti-Mouse antibody labeled with 6 nm colloidal gold (1:100, 115-195-146, Jackson Immuno Research) for 1 h. After sequentially washing with ddH2O twice and 2% w/v uranyl acetate, the grids were stained with 2% w/v uranyl acetate for 45 s. The excess buffer was removed with filter paper. The sample was dried with infrared lamp, and EM Images were acquired as described above. Immunogold negative stain EM confirmed that the purified fibrils were indeed Tau fibrils (Fig. 1).

Immunohistochemistry staining

For the histopathological studies, the mice were anaesthetized with over-dosed sodium pentobarbital (100 mg/kg, intraperitoneally) and transcardially perfused with ice-cold 4% paraformaldehyde (PFA). The mice brains were then collected and post-fixed for 3 days in 4% PFA, followed by a gradient dehydration in sucrose/PBS solutions (10%, 20%, 30%). Next, the mouse brains were rapidly frozen with OCT embedding compound (Sakura) and serially sectioned with a cryostat microtome (Leica).

Immunohistochemistry was performed as described previously, with slightly modification[4]. The serial brain sections (25 um) were collected and subjected to 3% hydrogen peroxide solution to quench its endogenous peroxidases. After blocked with 5% BSA/PBS plus 0.3% Triton X-100, sections were incubated with a primary antibody against p-Tau (1:500, MN1020, Invitrogen) for 16 h at 4°C. Then the sections were sequentially incubated with a biotin-conjugated secondary anti-mouse antibody (1:1000, BA-9200, Vector) for 2 h and avidin-biotin complex (1:1000, PK-6100, Vector) for 1.5 h at RT. After labelling using a DAB-peroxidase substrate (SK-4100, Vector), the sections were counterstained with hematoxylin. Images were captured by the DP74 digital camera connected to an Olympus microscope (Olympus).

Cryo-EM data collection

4 μl of the sarkosyl-insoluble fractions were applied to glow-discharged holey carbon grids (C-Flat CuR1.2/1.3, 300 mesh) and then plunge-frozen in liquid ethane after blotted with filter paper using Vitrobot Mark IV (FEI Company) at 100% humidity and 16° C. Cryo-EM images were acquired using Krios™ G4 cryo transmission electron microscope (Thermo Scientific™) operated at 300 kV with a BioContinuum™ K3 direct detector (Gatan, Inc.) in super-resolution mode. Inelastically scattered electrons were removed by a GIF Quantum energy filter (Gatan, Inc.) using a slit width of 20 eV. 40 frame movies per micrograph were recorded at ×105,000 magnification with a pixel size 0.83 Å $pixel^{-1}$. The total dose is ~55 e^{-} $Å^{-2}$ for a total exposure time of 2.0 s. For defocus values, a range of -1.0 to -2.4 μm was adopted. Automated cryo-EM data collection was performed by using EPU software (Thermo Scientific™).

Image pre-processing and helical reconstruction

40 movie frames per micrograph were corrected for beam-induced motion, aligned, dose-weighted, and further binned with a physical pixel size of 0.83 Å using MotionCorr2 1.2.1[5]. CTFFIND-4.1.8 was used to estimate the contrast transfer function of every micrograph [6]. Fibrils were manually picked using the "Manual picking" program in RELION 4.0[7].

Segments for reference-free 2D classification were individually extracted with a box size of 1024 pixels (with a pixel size 0.83 Å $pixel^{-1}$) and an inter-box distance of 84.992 Å and rescaled to 440 pixels. Segments were separated by reference-free 2D classification steps with a decreasing in-plane angular sampling rate (8°, 2°, and 0.5°) and a T=2 regularization parameter. Segments contributing suboptimal 2D class averages were discarded. The apparent half pitches and initial helical twist angles were calculated through the splicing of 2D class averages. Purified segments that comprise the entire helical crossover were used to construct 3D initial de novo models by the relion_helix_inimodel2d program[7].

Segments purified from 2D classifications were further extracted using box size of 360 pixels (with a pixel size 0.83 Å $pixel^{-1}$) without rescaling. 3D classifications (k=1) built a detailed map with these segments and the 3D initial models that were low-pass-filtered to 60 Å. An improved reference model served as the basis for a subsequent 3D classification (k=3). The higher resolution single class from this classification was then utilized for another 3D classification (k=1). Local search of symmetry to optimize the helical twist and rise was carried out after separation of β-strands was shown. We then performed 3D auto-refinements with optimization of helical twists and rises. The final reconstruction map was sharpened in using "Post-processing" program with a soft-edge solvent mask[7]. Overall resolution estimation was calculated based on the gold-standard 0.143 Fourier shell correlation (FSC) between the two independently refined half-maps. Local resolution was estimated using the "Local resolution" program in RELION 4.0 with the same mask and B-factor in post-processing[7].

Model building and refinement

According to the density map after post-processing, the atomic model of rTg4150 tau fibrils were built based on map and structure model of GPT fold as previously reported in COOT (PDB:7P6A)[8]. Then the models were refine against the corresponding map by phenix.real_space_refine program in PHENIX 1.13 with rotamer, Ramachandran, and geometry restraints[9]. There are more additional details in **Supplementary Table S1**.

Supplementary Figures

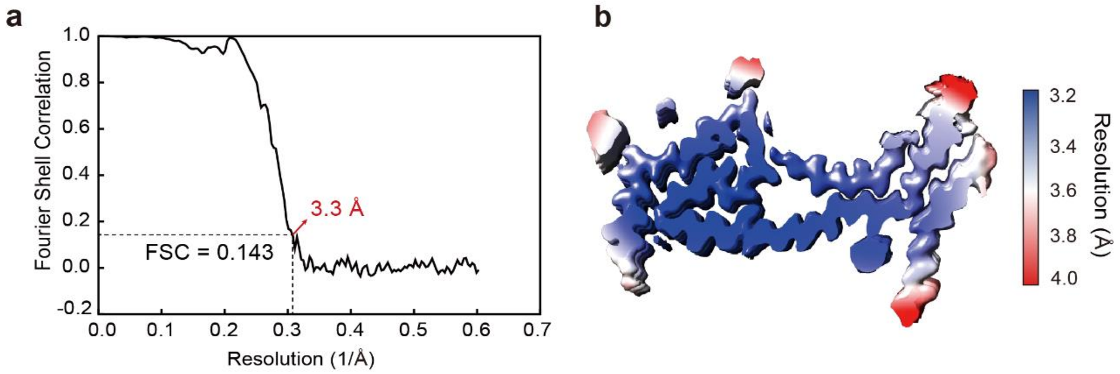

Figure S1. Local resolution estimation of the cryo-EM density map of rTg4510 Tau fibril. Gold-standard Fourier shell correlation (FSC) curve (**a**) and local resolution estimation of the reconstruction for the rTg4510 Tau fibril (**b**). The overall resolution of the rTg4510 Tau fibril is 3.3 Å.

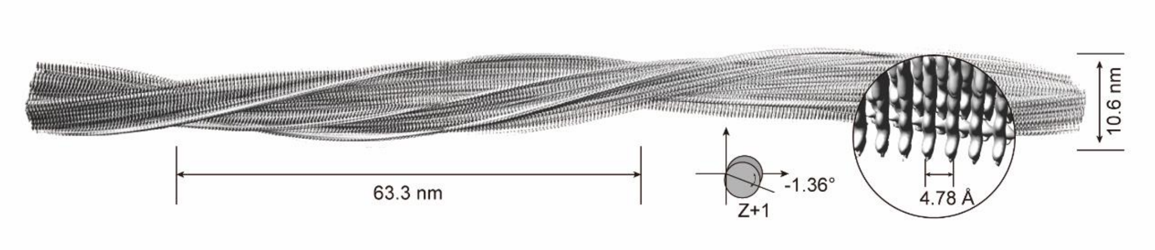

Figure S2. Cryo-EM reconstruction density map of the rTg4510 Tau fibril. Fibril width, length of half pitch (180° helical turn), helical rise, and twist angle are indicated.

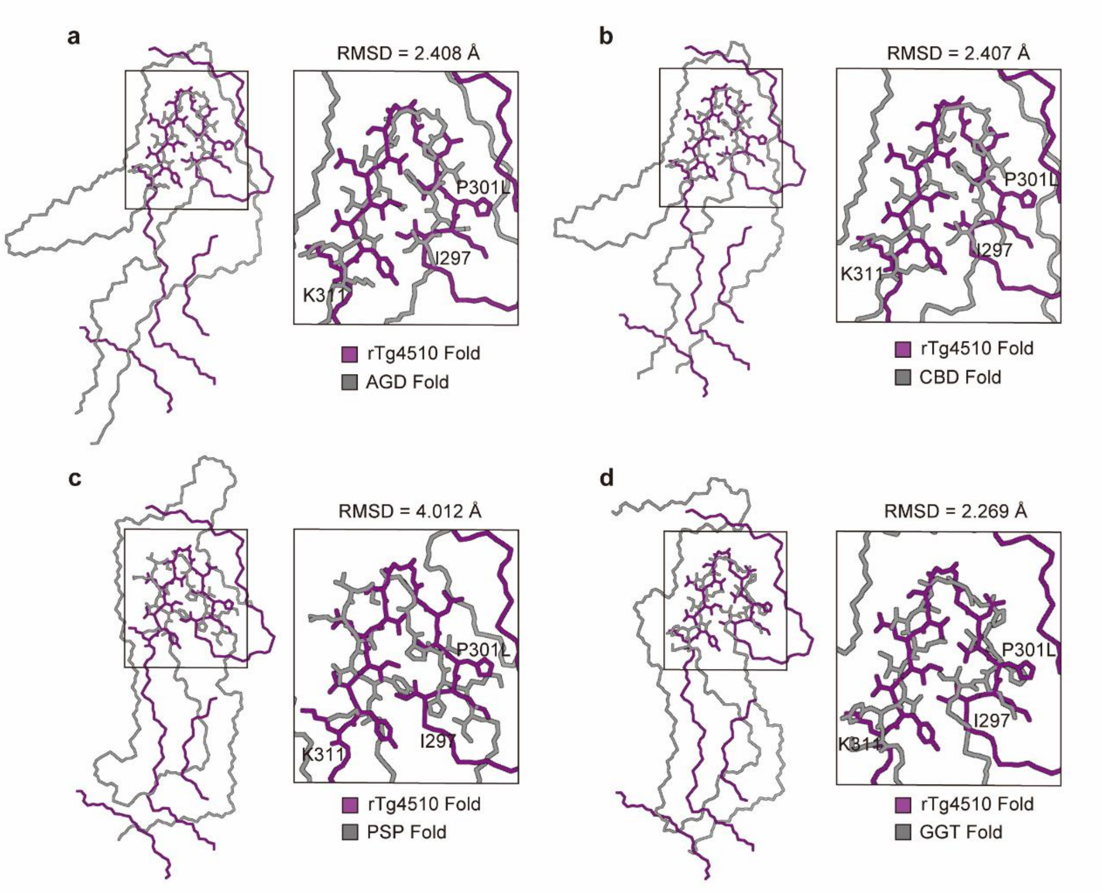

Figure S3. Structural comparison of the rTg4510 Tau fibril with human brain extracted Tau fibrils. Structural comparison of the rTg4510 Tau fibril with AGD fold (PDB:7P6D, **a**), CBD fold (PDB:6VHA, **b**), PSP fold (PDB:7P65, **c**), and GGT fold (PDB:7P66, **d**). The region used for comparison (residues 297-311) is zoomed in on the right panel with local RMSD indicated.

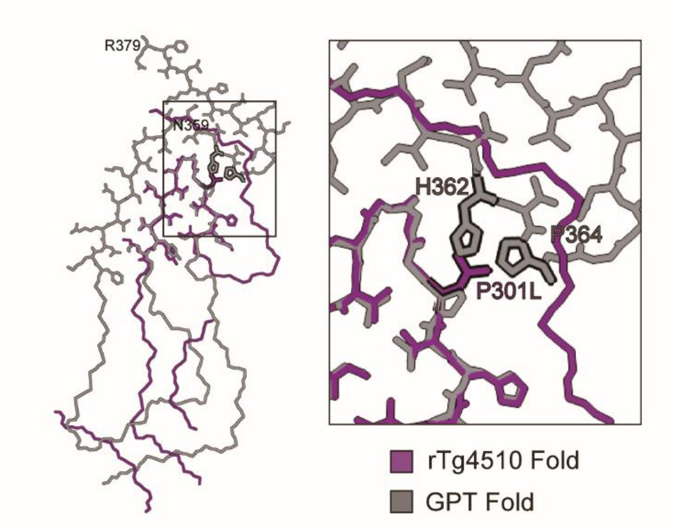

Figure S4. The P301L mutation clashes with H362 and P364 in the GTP fold. The superimpose of rTg4510 fold and GPT fold is on the left panel. Residues in clash are outlined in black and zoomed in on the right panel.

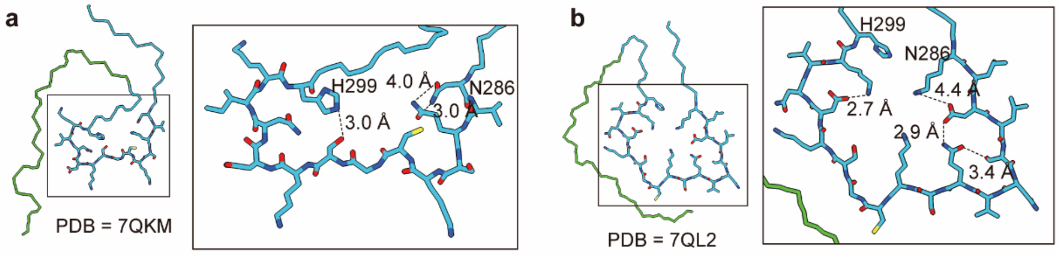

Figure S5. Hydrophilic interactions promote the folding within R2 region in WT Tau. Two polymorphs of Tau fibrils assembled *in vitro,*7QKM (**a**) and 7QL2 (**b**). The turns within R2 region are zoomed in with distances of hydrophilic interactions indicated.

Supplementary Table

Table S1. Cryo-EM structural determination and model statistics

Data collection and processing	**rTg4510 Tau fibril** (EMD- 37445) (PDB 8WCP)
**Data Collection**	
Magnification	105,000
Pixel size (Å)	0.83
Defocus Range (μm)	-1.0 to -2.4
Voltage (kV)	300
Camera	BioContinuum K3
Microscope	Krios G4
Exposure time (s/frame)	0.05
Number of frames	40
Total dose ($e^-/Å^2$)	55
**Reconstruction**	
Micrographs	5,000
Manually picked fibrils	9,704
Box size (pixel)	360
Inter-box distance (Å)	29.88
Initial particle images (no.)	264,762
Final particle images (no.)	92,188
Resolution (Å)	3.28
Map sharpening B-factor ($Å^2$)	-112.933
Helical rise (Å)	-1.36
Helical twist (°)	4.78
**Atomic model**	
Non-hydrogen atoms	1,536
Protein residues	225
Ligands	0
r.m.s.d. Bond lengths	0.004
r.m.s.d. Bond angles	0.754
All-atom clash score	14.23
Rotamer outliers (%)	0%
Ramachandran Outliers (%)	0%
Ramachandran Allowed (%)	11.59
Ramachandran Favored (%)	88.41

References

1 Hutton, M. *et al.* Association of missense and 5'-splice-site mutations in tau with the inherited dementia FTDP-17. *Nature* **393**, 702-705 (1998). https://doi.org:10.1038/31508

2 Ramsden, M. *et al.* Age-dependent neurofibrillary tangle formation, neuron loss, and memory impairment in a mouse model of human tauopathy (P301L). *J Neurosci* **25**, 10637-10647 (2005). https://doi.org:10.1523/JNEUROSCI.3279-05.2005

3 Fan, Y. *et al.* Generic amyloid fibrillation of TMEM106B in patient with Parkinson's disease dementia and normal elders. *Cell Res* **32**, 585-588 (2022). https://doi.org:10.1038/s41422-022-00665-3

4 Shen, Y. *et al.* Propagated alpha-synucleinopathy recapitulates REM sleep behaviour disorder followed by parkinsonian phenotypes in mice. *Brain* **143**, 3374-3392 (2020). https://doi.org:10.1093/brain/awaa283

5 Zheng, S. Q. *et al.* MotionCor2: anisotropic correction of beam-induced motion for improved cryo-electron microscopy. *Nat Methods* **14**, 331-332 (2017). https://doi.org:10.1038/nmeth.4193

6 Rohou, A. & Grigorieff, N. CTFFIND4: Fast and accurate defocus estimation from electron micrographs. *J Struct Biol* **192**, 216-221 (2015). https://doi.org:10.1016/j.jsb.2015.08.008

7 Zivanov, J. *et al.* A Bayesian approach to single-particle electron cryo-tomography in RELION-4.0. *Elife* **11** (2022). https://doi.org:10.7554/eLife.83724

8 Emsley, P., Lohkamp, B., Scott, W. G. & Cowtan, K. Features and development of Coot. *Acta Crystallogr D Biol Crystallogr* **66**, 486-501 (2010). https://doi.org:10.1107/S0907444910007493

9 Adams, P. D. *et al.* PHENIX: a comprehensive Python-based system for macromolecular structure solution. *Acta Crystallogr D Biol Crystallogr* **66**, 213-221 (2010). https://doi.org:10.1107/S0907444909052925
